# Sudden Cardiac Death due to Coronary Artery Vasculitis in a Patient with Relapsing Polychondritis

**DOI:** 10.1155/2020/5620471

**Published:** 2020-11-16

**Authors:** Heather Bukiri, Steven M. Ruhoy, Jane H. Buckner

**Affiliations:** ^1^Department of Rheumatology, Virginia Mason Medical Center, Seattle, WA, USA; ^2^Department of Pathology, Virginia Mason Medical Center, Seattle, WA, USA; ^3^Translational Research Program, Benaroya Research Institute at Virginia Mason, Seattle, WA, USA

## Abstract

Relapsing polychondritis (RP) is a systemic autoimmune disease characterized by relapsing and remitting inflammation of the cartilaginous structures of the ears, nose, tracheobronchial tree, and joints. Diagnosis is challenging due to the heterogeneity of clinical manifestations, the relapsing and remitting nature of the disease, the presence of coexistent diseases in at least one-third of patients, and the lack of a diagnostic blood test. Although RP-associated cardiac disease is the second most common cause of death behind tracheobronchial complications, coronary artery vasculitis is rare. This report describes a case of sudden cardiac death due to vasculitis affecting the coronary arteries in a patient with RP. The pathologic findings included obliterative coronary arteritis with plasma cells and storiform fibrosis, features suggesting that IgG4-related disease (IgG4-RD) may have contributed to the patient's cardiac disease. The literature on vasculitis and cardiac disease in RP and the possible role of IgG4-RD in this setting is also reviewed. The primary take-home message from this case report is the importance of frequent screening for cardiac disease, regardless of symptoms, in patients with RP. In addition, considering the diagnosis of IgG4-RD in some cases thought to be RP may also be warranted.

## 1. Introduction

Relapsing polychondritis (RP) is a rare autoimmune disorder characterized by relapsing and remitting inflammation of the cartilaginous structures of the ears, nose, tracheobronchial tree, and joints. Proteoglycan-rich structures of the eyes, heart, and vasculature are also commonly affected. Diagnosis is based on the presence of clinical features including bilateral auricular chondritis, nonerosive, seronegative inflammatory polyarthritis, nasal chondritis, ocular inflammation, respiratory tract chondritis, and cochlear and/or vestibular dysfunction as well as histology from biopsy of an inflamed tissue when available. The modified criteria for diagnosis require either three of the clinical features or one or more of these features plus confirmatory biopsy or chondritis at two or more separate anatomic locations with a response to glucocorticoids [1, 2]. Diagnosis is challenging due to the heterogeneity of clinical manifestations, the relapsing and remitting nature of the disease, the presence of coexistent diseases in at least one-third of patients, and the lack of a diagnostic blood test [3, 4]. The most common comorbidities are systemic vasculitis, other autoimmune diseases, and myelodysplastic syndrome. Current laboratory tests are nonspecific including antinuclear antibodies, anti-type II collagen (CII) antibodies, and antineutrophil cytoplasmic antibodies (ANCA).

Here, we present a case of sudden cardiac death due to vasculitis affecting the coronary arteries in a patient with RP. The pathologic findings included obliterative coronary arteritis with plasma cells and storiform fibrosis, suggesting that IgG4-related disease (IgG4-RD) may have contributed to the patient's cardiac disease. We also review the literature on vasculitis and cardiac disease in RP and the possible role of IgG4-RD in this setting.

## 2. Case Report

We describe a 55-year-old man with RP who died from apparent sudden cardiac death. He was diagnosed with RP at age 34, however his symptoms dated back to the age of 20. The patient developed saddle nose deformity at age 22, but was only diagnosed with RP when he developed recurrent auricular chondritis at the age of 34. His disease was further characterized by intermittent hoarseness, costochondritis and small joint arthritis. Laboratory evaluations included normal blood counts and chemistry panels, elevated ESR during flares, negative rheumatoid factor, the absence of antinuclear antibodies or ANCA and the presence of CII antibodies. In addition, we previously identified CII-specific T cells in this patient [5]. Over the course of his disease, the patient had been treated with methotrexate for 7 years, etanercept for 9 months, mycophenolate mofetil for 1 year, leflunomide for 2 years, and minocycline for 1 year. Prednisone and etodolac were used as needed during flares, which occurred 2-3 times per year. At the time of death, his disease had been well-controlled on subcutaneous adalimumab for many years. Notably, records of pulmonary function testing and transthoracic ultrasound evaluation of the heart and great vessels which are performed in routinely in many RP patients were not available for review. RP has recently been classified into three clinical phenotypes: mild, hematologic, and respiratory [6]. Based on our patient's clinical history, his clinical phenotype would have been classified as mild compared to the more severe hematologic and respiratory subtypes. Aside from his RP, the patient was an active, healthy man without history of tobacco use or other cardiac risk factors.

**Figure 1:**
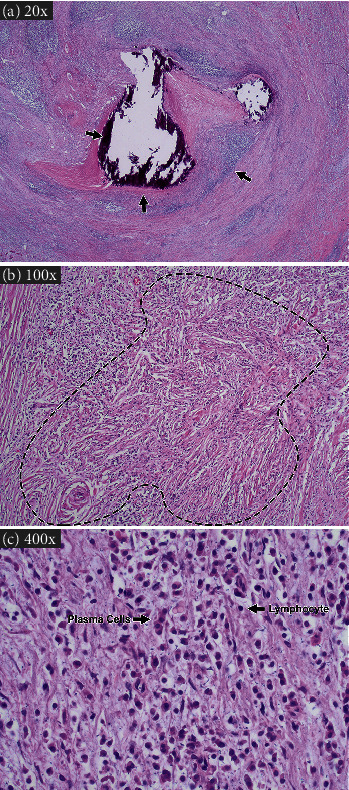
Histology of coronary artery from the patient described in this case report. (a) Low-power magnification of coronary artery demonstrating inflammatory infiltrate, hyalinization, and calcification (marked by arrows in clockwise fashion, respectively) (hemotoxylin and eosin (H&E), 20x). (b) High-power magnification demonstrating a storiform pattern of fibrosis, enclosed within the dashed line (H&E, 100x). (c) High-power magnification demonstrating lymphoplasmacytic nature of inflammation with arrows indicating representative plasma cells and lymphocytes (H&E, 400x).

An autopsy revealed that the heart was normal in size (490 grams) with a normal appearing aorta, left ventricle, epicardial surface, and valves. The coronary ostia, right circumflex, and left circumflex vessels were patent. The walls of the left main coronary artery and left anterior descending coronary artery were thickened, dull, white, and homogenous with partial to near total occlusion. Histologic examination of the coronary arteries revealed extensive infiltration of the intima and media by lymphocytes and plasma cells with obliterative, hyalinized fibrosis and calcification of the lumen (Figure 1). The internal elastic lamina was focally disrupted and predominantly lost. The inflammation and hyalinized fibrotic tissue extended into surrounding expanded adventitia and assumed a storiform pattern (Figure 1(b)). These findings were consistent with occlusive and obliterative coronary artery vasculitis, which was deemed the cause of death. Furthermore, the prominent storiform-type fibrosis was suggestive of IgG4-RD, although it was not possible to confirm this diagnosis as staining for IgG4-expressing plasma cells was not performed at the time of autopsy, and samples are no longer available for immunohistochemistry.

## 3. Discussion

RP-associated cardiac disease poses a high mortality burden, as it is the second most common cause of death behind tracheobronchial complications [4, 7]. Notably, cardiac involvement has been described in all three clinical phenotypes described by Dion et al., although it is most prevalent in the hematologic phenotype [6]. Risk factors for RP-related cardiac disease include older age and male sex, although it should be noted that women are also susceptible, with two reports of silent myocardial infarction in younger females [2]. Other cardiac manifestations include aortic vasculitis, aortic regurgitation and insufficiency, as well as less common manifestations such as aortic aneurysm or dissection, mitral valve prolapse or regurgitation, pericarditis, and myocarditis [2, 4, 8]. Conduction abnormalities, including atrioventricular block, supraventricular tachycardia, and paroxysmal atrial tachycardia, have also been described [4, 8]. Such arrhythmias estimated to occur in 4–6% of patients with RP, can be transient or permanent, and may require pacemaker implantation [8, 9]. Treatment of cardiac-related disease in RP can be challenging, as disease may be asymptomatic and progressive despite therapy [3, 10]. Combinations of corticosteroids, methotrexate, azathioprine, infliximab, and cyclophosphamide have been utilized with mixed success, while a recent publication describes successful use of tocilizumab for corticosteroid-resistant RP aortitis [8, 10–13]. When aortic insufficiency and coronary stenosis are severe, surgical treatment with aortic valve repair and coronary artery bypass grafting are indicated, respectively [4].

Coronary artery vasculitis is a rare cause of sudden cardiac death, estimated to account for 12% of nonatherosclerotic-related sudden cardiac death. It is typically associated with an underlying autoimmune or inflammatory condition such as RP, Takayasu arteritis, Kawasaki disease, Behçet's disease, IgG4-related periarteritis, eosinophilic granulomatosis with polyangiitis, granulomatosis with polyangiitis, and polyarteritis nodosa [14]. To date, there are only five case reports describing coronary vasculitis in RP (Table 1) [8, 11, 12, 15, 16]. Notably, cardiac disease was diagnosed during active RP in all four males but only during remission in the female. Furthermore, cardiac disease was diagnosed within 5 years of RP diagnosis in four of the five cases. One patient also experienced progression of cardiac disease despite improvement in symptoms and initiation of methotrexate and infliximab [16]. In contrast, the coronary vasculitis in our male patient was not preceded by any clinical history of great vessel or coronary disease and based on autopsy occurred in the absence of aortic disease. The cardiac event occurred years after diagnosis of RP and during a time of disease remission. Together, these cases underscore the importance for frequent screening for cardiac disease, regardless of symptoms, in patients with RP.

The prominent storiform-type fibrosis in the coronary artery of our patient was reminiscent of IgG4-RD, a chronic inflammatory condition affecting 2.2 per 100,000 individuals and occurring predominantly in middle-aged men. IgG4-RD can affect multiple organs and tissues, with affected tissues characterized by lymphoplasmacytic infiltration, storiform fibrosis, and obliterative phlebitis. IgG4 levels may also be elevated in the serum. Both arterial and venous vasculature can be affected by IgG4-RD, as aortic aneurysms, periaortitis, periarteritis, obliterative arteritis, and obliterative venous phlebitis have all been described [17]. Arteritis, periarteritis, and aneurysmal dilation of the coronary arteries have all been described with relation to IgG4-related cardiac disease. When the coronary arteries are involved, patients may be asymptomatic or minimally symptomatic but remain at risk for myocardial ischemia and sudden cardiac death [18].

**Figure 2:**
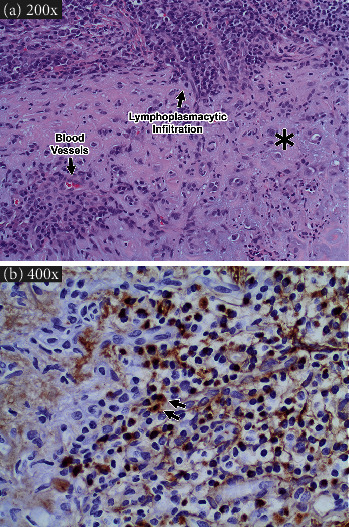
Histology of auricular cartilage, and immunohistochemical staining for IgG4 plasma cells from second patient with RP at our institute. (a) High-power magnification of auricular cartilage showing damage to cartilage including cartilage eosinophilia, infiltration by small blood vessels, as well as lymphoplasmacytic infiltration. The asterisk indicates cartilage eosinophilia, and arrows indicate representative small blood vessels and area of lymphoplasmacytic infiltration (H&E, 200x). (b) High-power magnification showing presence of IgG4-positive plasma cells (brown staining) in auricular cartilage, with arrows indicating representative IgG4-positive plasma cells (immunohistochemical staining of IgG4-positive plasma cells, 400x). There were 216 IgG4 + plasma cells and 601 IgG + plasma cells, and the IgG4/IgG ratio was 36%.

Given the rarity of both RP and IgG4-RD, it may seem unlikely for them to coexist; however, three patients have been described: a 67-year-old man with a 20-year history of RP who developed IgG4-RD involving his kidneys and pancreas [19], a 63-year-old man with a 1-year history of RP who developed IgG4-RD of the kidneys and lungs [20], and a 78-year-old man with clinical features of both RP and IgG4-RD [21]. In these cases, the RP predated the onset of IgG4-RD and manifested as auricular chondritis, ocular inflammation, and arthralgia. In addition, we are aware of two other cases suggesting a connection between IgG4 plasma cells and RP, although neither case met the IgG4-RD diagnostic criteria. Within our own institution, a 42-year-old man with psoriatic arthritis recently developed bilateral auricular chondritis and was diagnosed with RP. The auricular cartilage histology showed dense lymphoplasmacytic, cartilaginous, and perichondral inflammation characteristic of RP (Figure 2(a)). Notably, there was a high number of IgG4 plasma cells present albeit below the number required for IgG4-RD diagnosis (Figure 2(b); 216 IgG4 + plasma cells, 601 IgG + plasma cells, IgG4/IgG ratio = 36%). Horai et al. also presented a case of a 79-year-old man with RP with IgG4-expressing plasmacytes present in his left auricular cartilage [22].

These cases of RP and concomitant IgG4-RD suggest that the mechanisms that promote development of IgG4-RD and RP may be shared. A preferential IgG4 subtype of immunoglobulin response has been described in other autoimmune diseases such as pemphigus, muscle specific kinase myasthenia gravis, and thrombotic thrombocytopenic purpura [23]. Future studies should assess total and cartilage-specific IgG4 levels in RP. In addition, T-follicular helper cells could also be a driver of IgG4-RD and RP. Importantly, IgG4-RD can mimic some aspects of RP. Both diseases can lead to renal disease, tracheobronchial inflammation, and vasculitis, thus considering the diagnosis of IgG4-RD in some cases thought to be RP is warranted.

## 4. Conclusion

Cardiac disease and vasculitis in RP pose significant morbidity and mortality to patients. Such disease can be silent and occur even when other disease manifestations, such as chondritis, are inactive. To evaluate for cardiac involvement, patients should routinely be asked about chest pain, dyspnea, palpitations, presyncope, and syncope. Regardless of symptoms, screening for cardiac disease in patients with RP is recommended.

## Figures and Tables

**Table 1:** Comparison of our patient with five published case reports describing RP with concomitant coronary artery disease.

Reference	Age (yrs.)/sex	RP manifestations	Therapy prior to cardiac disease	Cardiac manifestations (duration of RP at the time of onset)	RP disease activity at time of cardiac diagnosis	Therapy	Response to therapy/clinical outcome
This case report	55/M	Auricular chondritis Nasal deformity Polyarthritis Respiratory chondritis	Adalimumab	Obliterative coronary arteritis	Well-controlled	Methotrexate Etanercept Mycophenolate Mofetil Lefulunomide Minocycline Prednisone Etolac	Sudden cardiac death

Bowness et al. 1991 [8]	33/M	Auricular chondritis Nasal collapse Polyarthritis Respiratory chondritis	Prednisolone (10 mg/day)	Complete heart block (4 wks.) Acute aortic incompetence (8 wks.) Coronary vasculitis detected postmortem	Active: elevated CRP, right auricular chondritis, inflamed vocal cords, hoarseness	Pacemaker AVR Prednisolone (60 mg/day) Cyclophosphamide (oral, 2 mg/kg/day)	Fatal acute heart failure (5 weeks postop.)

Vaidynathan et al. 2006 [12]	26/F	Auricular chondritis Nasal bridge depression	Prednisolone (10 mg/day for 1 year)	Severe aortic regurgitation (2 yrs.) Anterior and lateral wall myocardial infarction with right and left coronary artery ostial stenosis (34 mos.)	Inactive	AVR Prednisolone (20 mg/day)	Fatal cardiac arrest awaiting coronary artery bypass grafting

Stein et al. 2008 [11]	30/M	Auricular chondritis Nasal chondritis Uveitis	Prednisone (30 mg/day for 3 weeks)	Asymmetric proximal aorta and aortic root wall thickening, stenosis of ostium of left and right main coronary arteries, right external iliac artery stenosis (5 yrs.) Positive cardiac stress test (5 yrs.) Critical coronary stenosis (6 yrs.)	Active: auricular chondritis Normal inflammatory markers	AVR CABG Prednisone (1 mg/kg/day) Cyclophosphamide (oral, 100 mg/kg/day) Infliximab (5 mg/kg/dose)	Improvement in symptoms following surgery

McCarthy and Cunnane 2010 [15]	45/M	Auricular deformity Nasal deformity Arthralgia Bilateral sensorineural deafness	Methotrexate Azathioprine Cyclosporine A Infliximab Corticosteroids Rituximab	Severe aortic incompetence, normal aortic root, stenosis of main left and right coronary arteries (16 yrs.)	Active: elevated CRP	AVR CABG Azathioprine	Clinical remission after 18 mos.

Sugrue et al. 2014 [16]	51/M	Auricular chondritis Arthritis Aortic root dilation	N/A	Aortic root dilation (at time of diagnosis) Femoral bruit (4 mos.) Critical left main stem ostial stenosis (8 mos.) Progression of aortitis (36 mos.)	Active: elevated inflammatory markers that became quiescent with cardiac disease progression	Prednisolone (1 mg/kg) MTX (25 mg/wk) Infliximab (IV 5 mg/kg, 6 weekly initially, increased to 10 mg/kg, 6 weekly)	Normalization of inflammatory markers Asymptomatic after 12 mos.

AVR, aortic valve replacement; CABG, coronary artery bypass grafting; MTX, methotrexate.
